# Roles of mitochondria in the hallmarks of metastasis

**DOI:** 10.1038/s41416-020-01125-8

**Published:** 2020-11-04

**Authors:** Adam D. Scheid, Thomas C. Beadnell, Danny R. Welch

**Affiliations:** 1 Department of Cancer Biology, University of Kansas Medical Center, Kansas City, KS USA; 2 Heartland Center for Mitochondrial Medicine, Kansas City, KS USA; 3 University of Kansas Cancer Center, Kansas City, KS USA

**Keywords:** Cancer epigenetics, Cancer metabolism

## Abstract

Although mitochondrial contributions to cancer have been recognised for approximately a century, given that mitochondrial DNA (mtDNA) is dwarfed by the size of the nuclear genome (nDNA), nuclear genetics has represented a focal point in cancer biology, often at the expense of mtDNA and mitochondria. However, genomic sequencing and advances in in vivo models underscore the importance of mtDNA and mitochondria in cancer and metastasis. In this review, we explore the roles of mitochondria in the four defined ‘hallmarks of metastasis’: motility and invasion, microenvironment modulation, plasticity and colonisation. Biochemical processes within the mitochondria of both cancer cells and the stromal cells with which they interact are critical for each metastatic hallmark. We unravel complex dynamics in mitochondrial contributions to cancer, which are context-dependent and capable of either promoting metastasis or being leveraged to prevent it at various points of the metastatic cascade. Ultimately, mitochondrial contributions to cancer and metastasis are rooted in the capacity of these organelles to tune metabolic and genetic responses to dynamic microenvironmental cues.

## Background

**Fig. 1:**
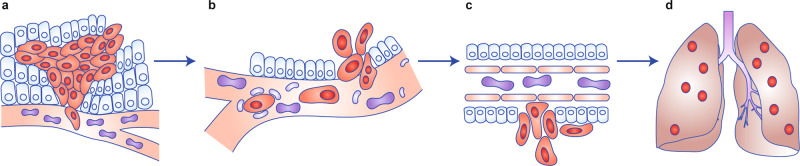
The metastatic cascade. Metastasis begins with cells acquiring the ability to invade surrounding stroma and enter the vasculature, lymphatics or coelomic cavity (**a**). In haematogenous metastasis, cells disseminate widely while interacting with other blood-borne cells and plasma components as well as with endothelial cells lining vessels (**b**). Upon arrest or attachment, tumour cells traverse the intimal layer and eventually the basement membrane (**c**) before proliferating to form discontiguous secondary foci in other organs (**d**). [Figure Adapted from ref. ^175^ with permission © DRW].

Thousands to millions of cells are shed from a neoplasm into the circulation every day, but the majority do not survive or result in a metastatic outgrowth.^1,2^ Disseminated cells are under extreme stress (from shear forces, the immune system, reactive oxygen species (ROS), nutrient deficiency, stemness, changing matrices), which greatly limits their ability to survive selective pressures during the journey from one organ to another (the metastatic cascade (Fig. 1)). Similarly, as human ancestors migrated north from Africa, they encountered new climates that required metabolic adaptations. Changing environments selected for mitochondrial DNA (mtDNA) variants that imparted metabolic advantages which, in turn, gave rise to distinct mtDNA haplogroups.

Mitochondria are believed to have evolved from an endosymbiotic relationship between an α-Proteobacterium (*Alphaproteobacteria*) living inside a eukaryotic host cell.^3,4^ Over millennia, mitochondria have evolved alongside nuclear (nDNA) such that mitochondria currently contain only 37 genes—22 tRNA, 2 rRNA and 13 electron transport chain (ETC) protein-coding genes—while all other genes encoding mitochondrial proteins are encoded in nDNA.^5^ At ~16,500 base pairs in humans and ~16,300 base pairs in mice, the mitochondrial genome is minute relative to its nuclear counterpart, leading many to ignore mtDNA in sequencing analyses.^6^ However, increasing numbers of studies demonstrating the relevance of mtDNA mutations in pathologies ranging from metabolic and musculoskeletal diseases to cancer underscore the importance of mtDNA in human health and disease.^6–9^ Early evidence that mtDNA and mitochondrial dysfunction could regulate metastasis came from Ishikawa et al., who transferred mitochondria from aggressively growing cancers into less aggressive cancers and observed an increase in aggressiveness.^10^ In addition, many mutations in ETC complex I components lead to increased levels of ROS, conferring an increased metastatic propensity.^10,11^ Notably, mutations in ND4 (C12084T) and ND5 (A13966G) in the MDA-MB-231 breast cancer cell line^12^ and missense and nonsense mutations in ND6 in the A549 lung cancer cell line^13^ increased experimental metastasis. Similarly, other mtDNA SNPs also alter metastasis efficiency in an oncogenic driver-dependent manner.^14^ In addition to serving as metabolic ‘powerhouses of the cell’, mitochondria have co-evolved with their hosts to serve as critical signalling hubs in several pathways. For example, mitochondrial signalling can influence cancer and metastasis in inflammation^15^ and apoptosis,^16^ as described below.

Discussing mtDNA and mitochondrial contributions to cancer and metastasis require a general understanding of the tools available (and unavailable) for interrogating mtDNA (see Box 1). Variations in mtDNA and nDNA occur in two forms: single-nucleotide polymorphisms (SNPs) and mutations. SNPs define mtDNA haplogroups and are inherited in a substantial portion of a population (at least 1% of populations^17^); whereas, somatic mutations occur spontaneously in individuals or cells. Both SNPs and mutations are relevant to cancer and metastasis, as reviewed in detail elsewhere.^18^ Some germline mtDNA SNPs predispose individuals to particular cancers (e.g. haplogroup N predisposes to breast and/or oesophageal cancers).^19–21^ Somatic mutations that arise after transformation can augment cancer and cancer progression. Mutation-mediated phenotypes are influenced by the genomic backgrounds in which they develop. SNPs and mutations whose effects are products of combinations with other alleles are called quantitative trait loci (QTL).^22^ QTL introduce a layer of complexity to genomic analysis, so that one often cannot attribute phenotypic alterations to single genes. We emphasise that mtDNA SNPs and mutations are unlikely to be solely responsible for differential susceptibility to cancer and metastases; rather, mtDNA SNPs act in concert with other nuclear and mitochondrial alleles as QTL.

The presence of genes encoding mitochondrial components in both nDNA and mtDNA not only necessitates mitochondrial–nuclear crosstalk to enable mitochondrial function but also creates a scenario in which intrinsic (mtDNA) and extrinsic (nDNA) mutations can influence mitochondrial function and—as discussed in this review—cancer and metastasis. Notably, owing to factors such as lack of histones, which when tightly compacted create a layer of protection for nDNA from damage induced by free radicals, mtDNA is more susceptible to mutation than nDNA. In addition, the proximity of mitochondria to reactive oxygen species (ROS) generated by oxidative phosphorylation further contributes to the estimated 10-fold higher mutation rate in mtDNA relative to nDNA.^23^

**Fig. 2:**
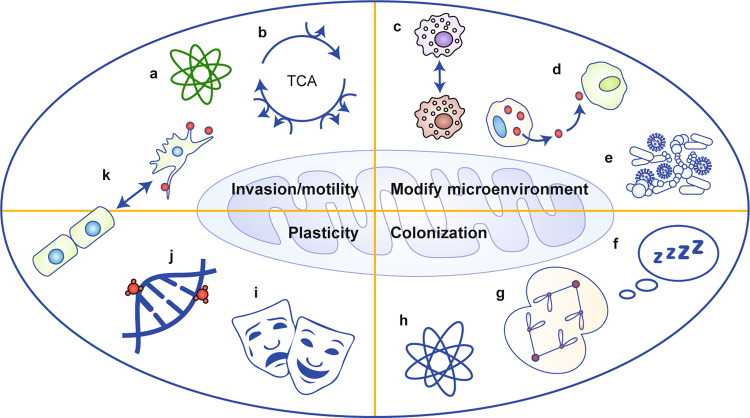
Mitochondrial contributions to the hallmarks of metastasis. The four hallmarks of metastasis—motility and invasion, modulation of microenvironments, plasticity and colonisation—are all impacted by mitochondrial functions, including: **a** reactive species (both oxygen and nitrogen); **b** catabolic metabolites; **c** immune cell polarisation or activation states; **d** secreted signalling molecules; **e** alteration of the microbiome; **f** regulation of cellular quiescence or dormancy; **g** regulation of cell division; **h** reactive species in the milieu of each tissue; **i** responses to stress; **j** epigenetic changes in cellular genomes; and **k** tumour cell transition states, such as EMT.

We postulate that metastatic efficiency requires the presence of mitochondria to help overcome the changing energetic selective pressures in metastatic microenvironments. As such, mtDNA SNPs and mutations might lead to selective differences in metastatic susceptibility in patient haplogroups and/or organotropism for certain cancer histotypes. To advance these ideas, we have consolidated the current literature on mitochondria in the context of the proposed hallmarks of metastasis:^1^ motility and invasion, modulation of the microenvironment, plasticity and colonisation. We aim to outline the complex, context-dependent contributions of mitochondria to each of the hallmarks (Fig. 2), We also discuss several remaining questions regarding mitochondrial contributions to cancer and metastasis, as well as currently existing challenges toward addressing those questions (See Box 1).

Box 1: Technical challenges to studying mitochondrial contributions in biological processes Studying direct contributions of mitochondria presents unique challenges due to distinct mitochondrial characteristics. (1) Editing mtDNA using traditional genetic engineering methods is difficult for three main reasons. First, localising entire desired mtDNA copies and in some cases editing machinery to mitochondria is not yet possible. Second, the failure to edit every mtDNA copy present in a cell produces mtDNA heterogeneity—called heteroplasmy—which confounds experimental interpretation. And third, mtDNA gene ‘lookalikes’ within nDNA complicate sequencing annotation.^176^ (2) Challenges associated with multiple variables. Given the occurrence of mitochondrial–nuclear crosstalk, isolating nDNA and mtDNA as independent experimental variables is also required to study mitochondrial contributions to cell biology but currently remains unethical in humans given a lack of knowledge regarding potential adverse effects. (3) Imperfect models. Notwithstanding the fact that all models fail to completely recapitulate the situation in people, experimental models to study mitochondria possess several unique caveats both in vitro and in vivo. Cytoplasmic hybrid—‘cybrid’— cells have been created by fusing nucleated cells with enucleated cytoplasts bearing the desired mtDNA.^177^ mtDNA depletion results in so-called rho-null (ρ^0^) cells into which replacement mtDNA can be introduced.^178^ Although powerful, methods generating ρ^0^ cells can affect nDNA. In mice, three main models have been developed (reviewed in refs. ^179,180^): transmitochondrial mice (also called ‘mito-mice’), conplastic mice and mitochondrial–nuclear exchange (MNX) mice. Transmitochondrial mice are generated using zygotic mitochondrial injection or ρ^0^ embryonic stem cells. Conplastic mice are generated by breeding female mice harbouring the mtDNA of interest with males bearing the nDNA of interest. Female progeny are repeatedly backcrossed with male mice containing paternal nDNA for at least 10 generations, resulting in the generation of conplastic mice with desired mtDNA and 99.9% desired nDNA.^181^ We generated MNX mice via oocyte pronuclear transfer.^182^ Pronuclear transfer provides advantages over other in vivo models owing to its lack of heteroplasmy and potentially confounding nDNA recombination.

Box 2: Changes in cancer cell metabolism Warburg hypothesised in the 1920s that oxidative phosphorylation is irreversibly defective in neoplasia, forcing cancer cells to rely on glycolysis despite the presence of oxygen (aerobic glycolysis).^183^ Although impaired oxidative phosphorylation can indeed lead to aerobic glycolysis in cancer cells,^184,185^ most cancer cells still retain the capacity for oxidative phosphorylation.^186^ Rather, glycolysis benefits tumour cells through rapid ATP production,^187^ macromolecule generation^188^ and survival under hypoxic conditions.^189^ So, although Warburg was correct in determining an importance for glycolysis in cancer, the interpretation is more nuanced—most cancer cells are proficient in using both glycolysis and oxidative phosphorylation. In several cancer types, oxidative phosphorylation is upregulated and might serve an oncogenic function.^190–194^

## Hallmark #1: motility and invasion

During the initial stages of metastasis, primary cancer cells receive signals to become more migratory and invasive. We have highlighted in this section many of the selective pressures, which impinge upon cancer cells and how those pressures impact the mitochondria and in return how mitochondria respond and facilitate cancer cell migration and invasion.

### Hypoxia and glycolysis

**Fig. 3:**
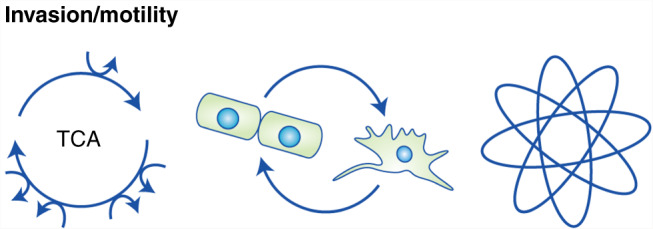
Motility and invasion. Invasion and motility are influenced by mitochondrial metabolic outputs, either from oxidative phosphorylation (left) or glycolysis. Motility and invasion exhibited by transformed epithelial cells is often associated with epithelial-to-mesenchymal transition (EMT, centre). EMT occurs at the leading edges of tumours, and results from gene expression changes in response to shifting dynamics in the tumour microenvironment. Reactive oxygen species (right), which can serve as signalling molecules, also alter tumour cell motility and invasion.

One well-characterised parameter that promotes the genetic reprogramming required for metastasis is hypoxia—in particular, the transcription factor hypoxia-inducible factor (HIF)-1α.^24,25^ Hill and colleagues were the first to show that hypoxia can increase metastasis,^25^ a finding subsequently verified by other teams.^24,26–28^ Hypoxic regions within tumours result from the demand for oxygen outpacing the supply. Hypoxia can also be intermittent which can add complexity to any interpretation, especially when coupled with differences in oxygen saturation within different regions of the tumour microenvironment.^29–31^ HIF-1α serves as an important sensor and regulator of cellular oxygen levels; it protects cells from undergoing apoptosis under hypoxic conditions as well as promoting glycolysis alongside corresponding decreases in mitochondrial oxidative phosphorylation in cancer cells (an effect known as the Warburg effect;  See Box 2). Most cancer cells are, however, proficient in using both glycolysis and oxidative phosphorylation (Fig. 3, left panel).

### Mitochondrial intracellular localisation

Cell motility and invasion take advantage of the localised generation of ATP by mitochondria to turnover of focal adhesions (FAs; a key component in cell motility) and actin cytoskeleton reorganisation (Fig. 3, centre panel). Neuronal axons served as a useful model for some of the first mitochondrial localisation studies, partly because axon processes are so far from the cell body that passive diffusion of ATP cannot fulfil the energy demands indicating that ATP must be synthesised locally, and partly because the active growth cone is a region of high ATP consumption.^32^ While studies in the early 1990s supported similar mitochondrial localisation in less elongated epithelial cells,^33,34^ only over the past 5 years have technological advancements made it possible to link similar metabolic pathways in mitochondrial localisation in neurons and cancer cells. Using a shRNA screen, Dario Altieri’s group demonstrated that many gene products linked to cytoskeletal trafficking in neurons (such as KIF5B, RHOT1 (miro), TRAK and SNPH) also participate in localising and concentrating mitochondria to the cell periphery of invading cancer cells—presumably to fuel the necessary energy demands.^35^ The same group has also identified and characterised mitochondrial proteins regulating cancer cell invasion/migration and metastasis, details of which can be found in refs. ^36–38^

### Reactive species

FAs are regulated partly by reactive molecular species or free radicals, including ROS and reactive nitrogen species (RNS) generated by the ETC in the process of mitochondrial oxidative phosphorylation (Fig. 3, right panel), which activate and induce the autophosphorylation of focal adhesion kinase (FAK). Although ROS and RNS are distinct, they are often collectively referred to as ROS and, for simplicity, we will do the same. ROS play important roles in cancer cell signalling,^39^ proliferation^40^ and the regulation of apoptosis,^41^ as well as cancer cell invasion and migration.^42^ Integrin-mediated changes in mitochondrial function that result in an increase in ROS production constitute an important mechanism of migration.^43^ Increased levels of ROS then serve as important mediators (activators) of Src and FAK signalling to regulate cell motility. It has also been observed that alterations in other proteins that regulate the production of ROS can also lead to increased cell invasion and metastasis.^44–46^ Of note, the mitochondrial deacetylase SIRT3 normally functions to repress ROS levels, thereby preventing increased Src oxidation. However, the loss of SIRT3 expression during cancer progression leads to increased migration through an increase in ROS levels, Src oxidation and FAK activation.^47^

### Cell death

The function of mitochondria as a hub for the regulation of programmed cell death is well known.^16^ Localisation of the pore-forming proteins Bak and Bax to the outer mitochondrial membrane (OMM) results in mitochondrial permeabilisation and the release of cytochrome *c*, downstream caspase activation and programmed cell death/apoptosis. Pro-survival proteins of the BCL-2 family prevent the accumulation of toxic Bax levels on the OMM by sequestering Bax.^48^ However, BCL-2 family members have been reported to have additional roles in invasion and migration.^49,50^ In addition to upregulating anti-apoptotic BCL-2 family proteins,^51^ tumour cells can evade apoptosis by limiting Bax access to OMM through mitochondrial hyperfragmentation.^52^ Subverting anoikis, the process by which epithelial cells die in response to lack of cell anchorage or anchorage to an unsuitable surface,^53,54^ is critical to metastasis. Metastatic cells can subvert anoikis through both cell autonomous^55^ and non-cell autonomous mechanisms.^56^

## Hallmark #2: modulation of the microenvironment

**Fig. 4:**
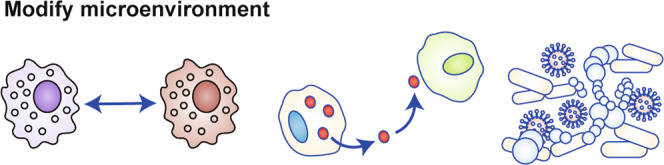
Modulation of the microenvironment. Tumour cells share complex interactions with other cells of the tumour microenvironment (TME) to enhance their own growth. One way tumour cells can facilitate tumour growth is by directing immune cells such as macrophages to differentiate into tissue remodelling, pro-tumourigenic subtypes (left). Tumour cells can also influence other cells in the TME via secretion of extracellular vesicles and tunnelling nanotubes containing mitochondria-derived molecules (centre). Conversely, tumour cells can receive mitochondrial signals from cells of the TME through the same mechanisms. Mitochondria-derived molecules can influence the microbiome in the TME and beyond, and the microbiome can influence tumour growth through metabolite production and inflammation induction (right).

For many years, the relative lack of cancer cell dependency on other cells led many researchers to mistakenly ignore the fact that cancer cells do not exist in a vacuum. As disseminated cells move throughout the body, the success or failure in establishing metastases is determined as much by landscapes in which they are found as the oncogenic drivers within the cell. The key components of this landscape include immune cells, fibroblasts, tissue-resident epithelial cells, endothelial cells and the microbiome, as well as interstitial fluids. Mitochondria are key mediators in the crosstalk that exists between tumour cells and the microenvironment. Just as mitochondria in tumour cells activate, interact with, and regulate the cells within the tumour microenvironment (TME) (Fig. 4), so too do mitochondria in the cells surrounding a tumour receive and interpret signals from tumour cells.

### Formylated peptides

Being derived from an ancient bacterial ancestor, mitochondria retain aspects of their bacterial lineage, such as N-terminal formylation of peptides and proteins. Similar to bacterial peptides, but not observed in eukaryotic nuclear-derived peptides, mitochondrial peptides begin with a formylated methionine. The release of these peptides during cancer cell death can be recognised by formyl peptide receptors (FPR) on innate immune cells (e.g. neutrophils), leading to an inflammatory response.^57^ These pattern recognition receptors, along with other receptors, respond to pathogen-associated molecular patterns (PAMPs) or damage-associated molecular patterns (DAMPs), which can lead to increased mitochondrial dysfunction, activation of the NLRP3 inflammasome and the secretion of pro-inflammatory cytokines.^58,59^ This results in inflammatory cell death termed pyroptosis. Activation of the immune system through these mechanisms and the promotion of inflammation can lead to increased vascularisation and the promotion of further influx of nutrients into the microenvironment which can stimulate cancer cell proliferation as well as provide an increased pathway for subsequent cancer cell migration across basement membranes.

### Reactive species

The production of reactive species by tumour cells is tightly linked to regulation of the TME. Immune cells generate anti-bacterial oxidants such as superoxide through NADPH oxidase, mitochondria and peroxisomes. ROS can be converted into more effective anti-bactericidal hydroxyl radicals through various enzymes including myeloperoxidase (MPO) or inducible nitric oxide synthase (iNOS). MPO is highly expressed in neutrophil granules and can convert hydrogen peroxide into hypochlorous acid.^60^ iNOS is typically expressed by M1 macrophages.^61^ The resultant nitric oxide (NO) has high affinity for iron bound by proteins and can inhibit the catalytic function of these proteins. High NO concentrations can also lead to increased DNA damage and, upon interaction with oxygen, can lead to the formation of peroxynitrite.^62^ Reactive species can also mediate cytotoxicity within the cell, and therefore cells are equipped with antioxidant mechanisms to counteract these intrinsic assaults. This balance between oxidants and antioxidants leads to important biological functions including cell signalling, fate determination and immune cell function.^63^ Changes in reactive species within the TME can have both positive and/or detrimental effects upon metastatic propensity. Cancer cells lacking antioxidant mechanisms may be eradicated due to these immune defence mechanisms while cancer cells that have evolved counteracting antioxidant mechanisms may receive increased survival signals and escape innate immune targeting.

### Immune cell polarisation

In addition to the production of cytotoxic reactive species, mitochondrial dynamics also play an important role in immune cell polarisation^64^ (Fig. 4, left panel). Highly proliferative cells tend to contain mitochondria that are undergoing fission, show a decreased production of ATP through oxidative phosphorylation, and rely more on glycolysis to obtain additional macromolecules.^64^ T-cell activation requires increased glucose and glutamine uptake^65–67^ and, consequently, decreased glucose levels reduce T-cell activation. This is especially important in the context of the tumour microenvironment, as T cells will be competing with tumour cells for resources.^68–70^ Importantly, constant tumour antigen exposure and T-cell stimulation can result in anergy, decreasing T-cell activation and reducing metabolic activity.^71^ In addition, studies have also defined roles for PD-1 in T-cell metabolic inhibition during T-cell exhaustion.^72,73^ T cells are reliant on mitochondrial metabolism for effective eradication of cancer cells. Tumour cell-mediated disruption in the ability of T cells to alter their metabolism will also likely facilitate an increased propensity for cancer cells to grow out within the TME.

Tumour cells, like activated T cells, are highly proliferative and often (but not always) harbour fragmented mitochondria.^74,75^ Anderson et al. demonstrated that mitochondrial hyperfragmentation in tumour cells represents a therapeutic vulnerability, where SMAC mimetics in particular can sensitise tumour cells with hyperfragmented mitochondria to apoptosis.^76^ As with other therapeutics targeting proliferative cells, a delicate balance must be struck wherein tumour cells are targeted without excessive immune cell targeting and potential immunosuppression.

Glycolytic metabolism largely polarises cells of both the innate and adaptive immune system towards being tumour inhibitory, while oxidative phosphorylation and fatty acid oxidisation (FAO) tend to polarise cells towards a tumour promotional or tissue remodelling phenotype. This can result in an increased propensity for cancer cells to evade immune recognition resulting in an increase in cancer metastasis. The role of mitochondria and metabolism in these cell types have been extensively reviewed elsewhere.^60,77–79^

### Cancer-associated fibroblasts and endothelial cells

Lisanti and colleagues have shown how cancer-associated fibroblasts (CAFs) influence the changing energetic needs of the TME. CAFs use glucose consumption to facilitate the production of high energy fuels such as pyruvate, lactate, fatty acids and ketone bodies, which are then used by cancer cells in the mitochondrial tricarboxylic acid (TCA) cycle to provide fuel (in what has been termed ‘the reverse Warburg effect’) for proliferation and to promote angiogenesis^80^ (Fig. 4, centre panel).

Endothelial cells also play important roles in the spread of cancer through their function in angiogenesis. The integrity of endothelial cell–cell adhesion is maintained by VE-cadherin, but the phosphorylation of VE-cadherin through a Rac-dependent signalling mechanism decreases cell–cell adhesion and increases blood vessel permeability. Importantly, this mechanism appears to be dependent on the production of mitochondrial ROS by Rac.^81^ Reduced blood vessel patency (and associated increased basement membrane exposure) is associated with increased metastatic efficiency.^82^

### Intratumoural interstitial fluid

Intratumoural interstitial fluid is the medium through which tumour cells manipulate other cells in the TME discussed above. Manipulation of TME cells by tumour cells through interstitial fluid components can be dynamic. For example, tumour cells can limit T-cell activation by depleting glucose required for T-cell activation from interstitial fluid.^70^ However, tumour cells can achieve the same end by leaving glucose levels unchanged but increasing interstitial fluid lactate levels.^83^ Heterogeneous tumour cell communication can leverage interstitial fluid as well. This is exemplified by metabolic symbiosis, where oxygenated tumour cells allow glucose to flow to less oxygenated tumour cells for aerobic glycolysis. In return, less oxygenated tumour cells return lactate to oxygenated tumour cells for pyruvate conversion and OXPHOS.^84^

### Microbiome

Another important facet of the microenvironment related to cancer and metastasis is the microbiome. The microbiome refers to the extensive populations of bacteria, archaea, protozoa, fungi and viruses that colonise organismal mucosal surfaces, and their associated genes.^85^ Although microbes other than bacteria are critical components of host physiology,^86,87^ we will focus exclusively on host mitochondria relationships with commensal bacteria that inhabit the intestines.

Commensal bacteria—in the intestines and other mucosal sites—outnumber epithelial cells by nearly 10:1. They are critical players in host physiology in health and disease,^88^ influencing cancer,^89^ metastasis^90^ and therapeutic outcomes.^91,92^ Bacterial roles in metastasis are well established in gastrointestinal cancers—indeed, data for gastrointestinal cancers are accordingly more abundant as intestines harbour more bacteria than other anatomical sites—but are probably influential in other cancer types as well. While balanced microbial profiles interact with host immunity to mediate immune tolerance and limit inflammation, dysbiotic microbial profiles can induce damaging inflammation to promote tumorigenesis and metastasis.^89,90^ Relationships between bacteria and host immunity can also influence immune-mediated responsiveness to therapeutic modalities, including chemotherapeutics^91^ and immunotherapies.^92^

The diversity of mucosal commensal bacteria is shaped by a complex and dynamic interplay of environmental and genetic factors.^93^ Among these factors, mitochondria and mtDNA influence commensal bacteria and vice versa^94^ (Fig. 4, right panel), which is intriguing, given the ancient bacterial ancestry of mitochondria. Through normal metabolism, bacteria produce short-chain fatty acids that can be converted into acetyl-coenzyme A (CoA) and TCA cycle intermediates in host-cell (i.e., both tumour and stromal cell) mitochondria.^95^ Consequently, microbial metabolic processing of host dietary substrates influences host mitochondrial metabolism. Conversely, oxidative phosphorylation and other mitochondrial metabolic processes in host cells produce ROS, which can be bactericidal.^96^ The cyclical nature of the host:microbial metabolic relationship underlies its complexity, and each aspect of the relationship can be co-opted by tumour cells. Tumour cell somatic mutations track with altered bacterial profiles in colorectal cancer (CRC), suggesting CRC metabolic by-products can select for colonisation of particular bacteria that—in return—enhance tumour growth.^97^ In short, an equilibrium is established between the tumour cells, stromal cells and the microbiome which, when disrupted, can alter tumour cell growth, metastasis and response to treatment.

mtDNA polymorphisms are another channel through which mitochondria influence the composition of commensal bacteria. Ibrahim and colleagues demonstrated, using a conplastic mouse model, that polymorphisms in the mtDNA ATP synthase 8 gene (MT-ATP8) correlate with altered intestinal bacterial profiles.^98^ Data from the Human Microbiome Project further support these findings, as the authors found correlations between altered bacterial populations and mitochondrial polymorphisms. Whereas the taxonomic profiles remained similar based on anatomical region in humans, bacterial species abundances changed based on mitochondrial haplogroup, and distinct bacterial population changes correlated with distinct mtDNA SNPs.^99^ Our MNX mouse work supports the observation that mtDNA influences commensal bacterial populations, as mice with identical nDNA but varying mtDNA display shifts in intestinal bacterial populations (paper in preparation). Work from Doug Wallace’s lab points towards a ROS-mediated mechanism to mediate the changes in commensal bacterial populations based on mtDNA SNPs^100^ (Fig. 4, right panel). An interesting alternative hypothesis is that mtDNA polymorphisms influence bacterial composition through immune selection, given that host mitochondrial formylated peptides are used to select T-cell repertoires recognising H2-M3, a non-classical MHC Ib molecule.^101^

Mitochondria are tightly linked between the many facets of the TME, controlling the ability of the cancer cells to extravasate, invade, and escape immune recognition to develop cancer metastases. In addition, mitochondria and mtDNA’s close linkages between bacterial ancestry may provide future clues to the role of the microbiome in the regulation of many of these processes.

## Hallmark #3: plasticity

**Fig. 5:**
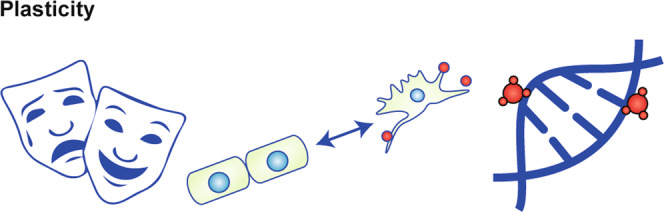
Plasticity. Metastatic cell plasticity refers to how neoplastic cells respond to inherent or microenvironmental stresses (e.g., hypoxia, ROS) and/or cues to change cellular states or behaviours (left). Epithelial-to-mesenchymal transition (EMT) and movement of mitochondria to leading edges of invading cells (red dots) exemplify mechanisms by which tumour cells adjust to altered conditions (centre). Simultaneous regulation of plasticity and tumour cell motility and invasion in the form of EMT demonstrate that mitochondria can influence more than one hallmark of metastasis using the same mechanism. Responses to dynamic metabolic demands in changing microenvironments is governed by mitochondrial sensing of extracellular signals that, in turn, result in mitochondria altering nuclear genome epigenetic marks (right).

Metastasis is a dynamic process and typically requires plasticity to ensure that the initial engagement of invasive and migratory genes is followed by the re-expression of proliferative genes upon reaching the metastatic site, to facilitate outgrowth (Fig. 5, left panel). The most studied plasticity mechanisms relate to the processes of epithelial-to-mesenchymal transition (EMT; Fig. 5, centre panel) and mesenchymal-to-epithelial transition (MET).^102^ Both processes involve alterations in gene expression that result from changing TMEs, hypoxia, immune infiltration or other interactions at the leading edge of the tumour. This section will focus on the current proposed roles of mitochondria in the regulation of changes at the epigenetic level. As the timeframe for transient signals to alter cellular behaviour rapidly does not involve cell division and fixation of mutations in the genome, the adaptations are epigenetic in nature. For further details please see the following review.^103^

### Mutations in the TCA cycle

A direct link between mitochondria and cell plasticity is demonstrated by mutations in the TCA cycle.^103^ Mutations in the mitochondrial enzyme fumarate hydratase (FH), which catalyses the reversible conversion of fumarate to malate, lead to increased levels of fumarate, which suppress anti-metastatic miRNAs and consequently cause the increased expression of EMT-related transcription factors.^104^ Similarly, mutations in succinate dehydrogenase (SDH) B have been associated with the increased expression of EMT-related genes, including the Snail and Slug transcription factors,^105,106^ and isocitrate dehydrogenase (IDH) mutations have been associated with the expression of the ZEB transcription factor family member Zeb1, which also promotes EMT.^107^

### Mitochondrial number and metabolic flexibility

Metabolic flexibility confers on tumour cells the ability to adapt to different conditions—and thus metabolic demand—in primary and metastatic microenvironments. One way to ensure metabolic flexibility is by changing the number of mitochondria in a given cell. Mitochondria are generated via mitochondrial biogenesis and eliminated by mitophagy.^108^ However, the relationships between mitochondrial biogenesis, mitophagy, tumorigenesis and metastasis are far from simple. Both mitochondrial biogenesis and mitophagy can be advantageous or disadvantageous at various points of transformation and cancer progression.

Mitochondrial biogenesis is regulated by peroxisome proliferator-activated receptor γ coactivator-1 α (PGC-1α). PGC-1α, by coactivating nuclear respiratory factors 1 and 2 (NRF-1 and NRF-2), induces the expression of transcription factor A, mitochondrial (TFAM).^109^ As the name suggests, TFAM serves as a transcription factor and is a master of mtDNA replication and repair.^110,111^ Mitochondrial biogenesis can serve dichotomous roles in cancer metastasis, depending on the dynamic metabolic requirements of metastatic cells. Kalluri and colleagues observed that increased PGC-1α expression correlates with invasiveness and metastasis in breast cancer, demonstrating that increased mitochondrial mass and oxidative phosphorylation benefit at least some aspects of metastasis.^112^ However, we showed that the metastasis suppressor KISS1 interacts with NRF-1 to increase PGC-1α expression and mitochondrial biogenesis, with a corresponding shift from aerobic glycolysis to oxidative phosphorylation in C8161 metastatic melanoma cells, but that the cells underwent a decrease in invasion and migration.^113^ It, therefore, appears that the association of higher PGC-1α levels and mitochondrial numbers with metastasis is likely to depend on the tissue.

Damaged and/or dysfunctional mitochondria in all cell types are eliminated from cells by mitophagy. One indication of mitochondrial damage is a lack of ability to generate the necessary proton gradients across the inner mitochondrial membranes (IMM) for ETC function. This depolarisation induces the phosphorylation of proteins on the OMM.^114,115^ OMM protein phosphorylation recruits and activates Parkin,^116^ which ubiquitinates OMM proteins to initiate degradation and to recruit autophagy adaptors.^117,118^ So, like mitochondrial biogenesis, mitophagy can engender metabolic flexibility, which can enable metastatic cell survival in changing microenvironments with dynamic metabolic demands. Mitophagy can also confer therapeutic resistance as demonstrated by Hu et al, who showed tumour cells can upregulate mitophagy mediators such as BNIP3 to adapt to hypoxia induced by anti-angiogenic therapies.^119^ On the other hand, mitophagy can induce apoptosis and halt proliferation in cervical and breast cancer cells, respectively.^120,121^ Overall, mitophagy provides a mechanism of plasticity wherein tumour cells can eliminate mitochondria— either damaged or normal but superfluous—to serve the dynamic metabolic needs of tumorigenesis and metastasis. A deeper understanding of how tumours manipulate mitophagy is paramount, as it may uncover therapeutic vulnerabilities.

### Mitochondria, nDNA gene expression and epigenetics

An important aspect of mitochondrial–nuclear crosstalk is the contribution of mitochondria to nDNA epigenetic landscapes and gene expression^122–125^ (Fig. 5, right panel). Vivian et al.^126^ demonstrated mitochondrial influence over nDNA modifications and gene expression by showing that MNX mice with identical nDNA but distinct mtDNA displayed selective nDNA methylation and gene expression differences.^126^

Mitochondrial contributions to nDNA epigenetics and gene expression are thought to be mostly mediated by mitochondrial metabolites. For example, acetyl groups for nDNA and histone acetylation can be acquired from acetyl-CoA, which is produced as a substrate for the TCA cycle in mitochondria.^127^ However, how changes in metabolite pools induce selective changes in nDNA epigenetics and gene expression is not fully understood. One explanation might be mitochondrial small non-coding RNA (sncRNA), as discussed below, but there are minimal data to support this hypothesis. Xie et al. used mtDNA-deficient (ρ0) prostate adenocarcinoma cell (LNCaP) and ρ^0^ human breast adenocarcinoma cell (MCF-7) models to demonstrate that mtDNA can influence the expression of DNA methyltransferase 1 (DNMT1),^128^ altering the methylation of genes that are relevant to cancer and cancer progression.^128^ For example, mtDNA deficiency induced DNMT1 expression, which led to the hypermethylation of the promoters for endothelin B receptor, O^6^-methylguanine-DNA methyltransferase and E-cadherin, ultimately silencing the expression of these genes. Hypermethylation is also known to mediate silencing of additional metastasis suppressor genes, such as BRMS1 and KISS1,^129,130^ suggesting that, in the case of KISS1, for example, mitochondria might influence metastasis suppressor gene silencing as well as metabolism.^113,131^ Clearly, more data are required to establish firm connections from these observations. Nevertheless, mitochondria are central to the response(s) of tumour cells to microenvironmental signals as well as the communication of tumour cells with the surrounding milieu.

### Mitochondrial sncRNA

In addition to changing metabolite pools (e.g. through altering expression of transcripts encoding mitochondrial metabolic proteins^132^), mitochondrial sncRNA represent a means by which mitochondria might selectively regulate the nuclear genome. In general, sncRNA studies focus on nDNA-encoded sncRNA, but multiple sncRNA classes are encoded within mtDNA as well.^133,134^ mtDNA-encoded sncRNA derives from intergenic regions from all three mtDNA gene types: tRNA, rRNA and protein-encoding genes.^134^ mtDNA-encoded sncRNAs that have been characterised include microRNA (miRNA),^135^ tRNA-derived fragments (tRF),^136^ rRNA-derived fragments (rRF),^137^ Piwi-interacting RNAs (piRNA)^138^ and long non-coding RNA (lncRNA).^139^ In addition to the ability of mtDNA-encoded sncRNA to influence the nuclear gene expression, nDNA-encoded sncRNA target nDNA-encoded mitochondrial mRNA and are imported into mitochondria to regulate mitochondrial function.^135^

As they do in normal physiology, mitochondrial sncRNAs regulate the responses to dynamic cellular microenvironments in cancer and metastasis. Mitochondrial sncRNAs can suppress or promote cancer and metastasis. miRNAs target multiple aspects of mitochondrial biology in cancer, including aerobic glycolysis, mtDNA transcription, apoptosis and metabolism.^140^ The expression of mtDNA- and nDNA-encoded tRFs varies according to multiple factors including cancer and cancer type, suggesting these sncRNAs function in cancer.^141^ Goodarzi et al. demonstrated that nDNA-encoded tRFs interact with the RNA-binding protein YBX1 and, in-so-doing, influence metastasis.^142^ Mitochondrial tRF—YBX1 interactions have not so far been described, but the interplay between tRFs and metastasis—alongside the localisation of mitochondrial tRFs in the cytosol^143^ and functional versatility^144^ —support the possibility that mitochondrial tRFs might also influence metastasis.

### Mitochondria-associated membranes

In addition to participating in nuclear crosstalk, mitochondria share dynamic, complex interactions with ER at so-called mitochondria-associated membranes (MAM). MAM comprise physical interactions between ER and mitochondria, in which protein tethers and spacers keep the organelles 15–30 nm apart.^145^ MAM have garnered particular interest in cancer biology, as numerous pro- and anti-tumorigenic proteins localise to the membranes.^146^ In addition, MAM facilitate Ca^2+^ exchange between ER stores and mitochondria, which is critical to several downstream cellular functions that impact cancer and metastasis. Mitochondrial sensitivity to ER Ca^2+^ release can be dynamically altered by changing the distance between ER and mitochondria, which is regulated by protein spacers.^147^ Under homoeostatic physiological conditions mitochondria serve as intracellular Ca^2+^ “buffers,” importing and exporting Ca^2+^ using ETC-generated membrane potentials and ion channels to influence metabolism, dictate cell fates, and keep cytosolic Ca^2+^ concentrations consistent.^148,149^ Some Ca^2+^ released from ER stores in response to cell signalling cues is imported into mitochondria at MAM, allowing mitochondria to serve the dynamic needs of the cell by upregulating ATP production.^150^

Mounting evidence indicates mitochondrial Ca^2+^ dysregulation plays roles in cancer and metastasis.^146,150^ Mitochondrial Ca^2+^ depletion has been associated with the Warburg effect in cancer cells, and, conversely, increased Ca^2+^ levels alongside increased ROS production has been observed in metastatic cells.^150^ Moreover, increased expression of an IMM Ca^2+^ importer—mitochondrial Ca^2+^ uniporter—is associated with breast cancer invasiveness and motility.^151^ As with ROS, metastatic cells strike a balance with increased mitochondrial Ca^2+^ levels, as mitochondrial Ca^2+^ overloading can induce cytochrome c release and apoptosis.^152^ The prevalence of mitochondrial Ca^2+^ regulation in pathophysiological conditions including cancer is being realised clinically, where peptides that can alter Ca^2+^ fluxes at MAM are being investigated.^153^

### Extracellular vesicles

Extracellular vesicles (exosomes and microvesicles) are membrane-bound vesicles of varying sizes and content that are released from cells to facilitate intercellular communication in diverse homoeostatic processes. EVs also mediate aspects of communication between tumour cells and other cells of the TME,^154^ and mitochondrial metabolites are a critical cargo of EVs. EVs derived from CAFs contain amino acids, lactate and acetate that can help push tumour cells towards aerobic glycolysis.^155^ Alternatively, CAF-derived EVs can provide tumour cells with TCA cycle intermediates for oxidative phosphorylation, demonstrating that CAF-derived EVs help to meet dynamic metabolic requirements during tumour growth.^156^ Metastatic breast cancer cells can acquire mtDNA from murine CAF-derived EVs which, in turn, promotes oxidative phosphorylation in the tumour cells.^157^ In addition, tumour-cell-derived EVs can alter metabolism in the TME. For example, EVs derived from colorectal carcinoma cells can induce aerobic glycolysis in adjacent non-transformed colonic epithelial cells to augment their growth.^158^ Medina et al. suggest that the packaging of mitochondrial metabolites into EVs is an intentional, active process, rather than a random, passive one.^159^ EVs containing entire mtDNA copies have been found in the circulation of metastatic breast cancer patients who are resistant to hormone therapy.^157^ It is possible that these EVs could be taken up by cancer cells with damaged mtDNA resulting in a restoration of a functional cancer cell mitochondrial metabolism and increased survival.

## Hallmark #4: colonisation

**Fig. 6:**
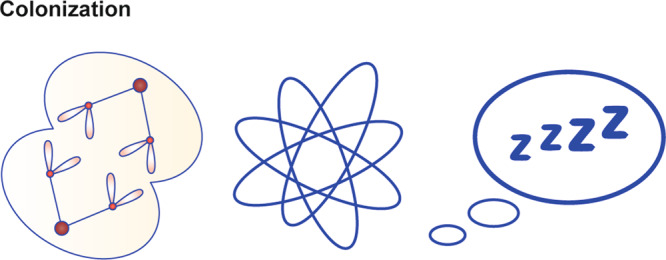
Colonisation. Metastatic cell colonisation is affected by changes altering the balance between cellular proliferation and apoptosis, and recycling cellular components (e.g., mitophagy and/or mitochondrial biogenesis, left). The ability of disseminated tumour cells to colonise new microenvironments is dependent in part on mitochondrially-derived products, including reactive molecular species (centre). Mitochondrial molecules can also influence organotropism and proliferation rates. This balance can produce dormant metastatic lesions that can persist in the absence of outgrowth for years (right).

Colonisation—the expansion of a disseminated cell in the metastatic microenvironment—is a unique dynamic described by Stephen Paget as ‘seeds growing in fertile soils’.^159^ Paget observed that the microenvironment selects cells that are most fit to survive in the metastatic environment. The capacity to colonise requires both a healthy/fit tumour cell and an amenable microenvironment,^160^ and colonisation is influenced by changes that alter the balance between proliferation and apoptosis, and mitochondrial biogenesis and mitophagy (Fig. 6, left panel).

As stated previously, mitochondria are more susceptible to mutation than the nuclear genome.^161^ It stands to reason, then, that decreased mitochondrial activity arising from such mutations would have detrimental effects on the ability of cells to survive the metastatic process. So how do cancer cells maintain mtDNA integrity under conditions of high stress such as those encountered during metastasis? Cells must maintain ROS levels in what is termed the hormetic zone or safe zone, as too much mtDNA damage can lead to toxic levels of ROS. Thus, the phenomenon of maintaining mitochondrial signalling molecules at safe levels—mitohormoesis—must come into play^162^ (Fig. 6, centre panel). Mechanisms through which tumour cells can maintain mitohormoesis are described below.

### The unfolded protein response

Analysis in breast cancer suggests that cancer cells upregulate the mitochondrial unfolded protein response (UPR^mt^) to deal with increasing mitochondrial stress. Although increasing levels of ROS promote increased invasiveness and metastasis, the increase is accompanied by increased UPR^mt^ to balance the toxicity and maintain cell viability.^163^ The endoplasmic reticulum (ER), with which mitochondria closely associate as described above, is an important regulator of this stress response. Evidence indicates that the ER promotes mitochondrial F-actin stabilisation through the actin-bundling protein fascin, which helps to maintain mtDNA stability via the promotion of oxidative metabolism.^164^ Fascin levels in some lung cancers are increased in advanced stages and have been shown to promote mitochondrial F-actin stability.^164^

### Mitochondrial transfer

Another way to restore mtDNA homoeostasis is through mitochondrial transfer, which probably occurs after mitochondria become irreversibly damaged. Berridge and colleagues tested this hypothesis by injecting either B16-F10 ρ^O^ melanoma cells or 4T1 ρ^O^ mammary carcinoma cells into mice; tumours still formed, but their formation was delayed. The injected cells regained mitochondrial respiration capacity, which correlated with tumorigenicity,^165^ by receiving host mitochondria via tunnelling nanotubes.^166^ Altered tumorigenic gene expression profiles from the replenishment of mtDNA varied according to the quantity (mtDNA copy) and the mitochondrial source (i.e., from other tumour cells, from specific stromal populations).^167^ That neoplastic cells can acquire mitochondria from various other cell types raises several questions. Could transferred mitochondria alter sensitivity to treatment? Mitochondrial transfer might well aid in evading the effects of chemotherapy and driving therapeutic resistance.^168–170^ This possibility is especially intriguing for chemotherapies that target mitochondrial ROS production in acute lymphoblastic leukaemia (ALL),^170^ but might also be relevant for more broad chemotherapies as demonstrated by mitochondrial transfer models in breast cancer.^168,169^ Another issue is whether mitochondria from specific tissue could confer organotropism. However, we know of no data either supporting or refuting this possibility.

Marlein et al. found that bone marrow stromal cells can transfer mitochondria to multiple myeloma cells, and that this transfer results in increased mitochondrial metabolism. Surprisingly in this study, the recipient cells acquired additional mitochondria despite containing functional mitochondria (calculated to be ~3% foreign mtDNA uptake). Interestingly, this transfer promoted an increased metabolic plasticity in the multiple myeloma cells, which allowed for the highly glycolytic myeloma cells to increase the use of OXPHOS in the presence of glycolytic inhibitors resulting in an increase in survival. The authors further demonstrated that myeloma cells have an increased dependence on the membrane glycoprotein CD38, and that mitochondrial transfer was partly regulated through a CD38-dependent mechanism.^171^

### Dormancy

Not all patients exhibit metastases upon primary tumour detection; disseminated cells can be dormant (Fig. 6, right panel), and a substantial portion of patients relapse months to years later. Long-term cancer cell survival at metastatic sites can go undetected for upwards of 10–15 years before colonisation eventually occurs. Autophagy plays a role in cell survival under conditions of low nutrients or stress, allowing cells to degrade and recycle cellular material. Likewise, autophagy/mitophagy has been demonstrated to play important roles in maintaining cell survival in dormant states.^172,173^ Dormant cells might also represent a population of relatively non-proliferative cancer stem cells. Similar to other less proliferative cell types, cancer stem cells tend to rely on oxidative phosphorylation and fatty acid oxidation.^174^ Dormancy allows cancer cells to avoid therapeutic strategies aimed at rapidly proliferating cells resulting in a potential future relapse. This again highlights an important survival mechanism maintained by a cancer cell’s ability to utilise glycolysis as well as OXPHOS.

## Discussion

After reviewing correlative data showing how mitochondria are associated with metastasis, several conclusions can be made. First, mitochondria can be both beneficial and detrimental to (potentially) metastatic cells. Second, mitochondrial gene products interact with other QTL to change metastatic efficiency. We have hopefully conveyed the relevance of mitochondrial genomes contributing to cancer metastasis. Like other quantitative trait loci, mitochondria are rheostats that adjust the scope and intensity of signals coming into a cell and emanating from the nucleus. Likewise, mitochondria can ‘polarise’ tumour cells and stromal cells, rendering them more or less pro- or anti-tumorigenic/metastatic. Metastatic tumour cells adopt properties that make them more motile; and, immune cells become less anti-tumorigenic and more helpful to cancer cells. Third, mitochondrial genetics and biochemical processes are associated with every step of the metastatic cascade and with all four of the proposed hallmarks of metastasis—motility and invasion, microenvironment modulation, plasticity and colonisation. The multiple ways in which mitochondria are involved in the hallmarks of metastasis are shown schematically in Figs. 2–6. While plasticity and adaptability are shown, the variations in metabolic and behavioural phenotypes are enormous and underrepresented by what is depicted in each figure.

However, many outstanding issues remain. Chief among them is the nature of the signals that mitochondria use to alter the nuclear genome, other cancer cells, stromal cells and the microbiome. We have described evidence for each of the signals although the jury is still out as to their identities.

Ultimately, we now know that mitochondria have a bigger role in metastasis than previously thought. In fact, the scope of mitochondrial contributions to metastasis is growing almost daily. Direct cause–effect relationships are still being worked out, but accumulating data continue to support multiple roles for mitochondria in cancer, especially in metastasis. As with any newly discovered relationship, the data are raising new and interesting questions. One aspect of mitochondrial genetic studies that we wish to emphasise is the paucity of robust models that yield unambiguous results. Each experimental model has strengths and limitations (See Text Box 1). As new experimental models and tools for directly comparing mtDNA sequences between species come online, the ability to cross-validate and extrapolate to other systems (e.g., mouse to human) will reduce experimental ambiguity. Nevertheless, our hope is that, with increasing awareness, others will join in the pursuit of mito-tastasis—the study of mitochondrial roles in cancer metastasis.

## Data Availability

Data and reagents are made available to the scientific community in accordance to policies of the University of Kansas Medical Center and the National Institutes of Health (USA).
